# Perception of synchronization in singing ensembles

**DOI:** 10.1371/journal.pone.0218162

**Published:** 2019-06-12

**Authors:** Sara D’Amario, Helena Daffern, Freya Bailes

**Affiliations:** 1 Department of Electronic Engineering, University of York, Heslington, York, United Kingdom; 2 School of Music, University of Leeds, Leeds, United Kingdom; Fred Hutchinson Cancer Research Center, UNITED STATES

## Abstract

Recent investigations analysing synchronization in singing ensembles have shown that the precision of synchronization during singing duo performances is better in the presence of visual contact between the singers than without. Research has also shown that synchronisation improves with practice across rehearsals in a newly formed singing quintet. However, whether listeners’ perceptions of synchronization reflect the different patterns of synchronization that were observed during ensemble performance with and without visual contact and across rehearsals has not yet been investigated. This study aims to analyse the perception of the synchronization associated with altered visual contact and amount of rehearsal during singing duo and quintet performances respectively, for listeners with different levels of musical expertise. A set of fifty-eight singing recordings selected from duo and quintet ensemble performances, collected from previous investigations of interpersonal synchrony, was presented to 33 listeners, including non-experts (university students with little or no music training), performers in the group (singers who performed the pieces used for the study), and other musicians (advanced music students). Participants were required to listen to each trial and judge the level of “togetherness” on a sliding scale from zero to 100. Results show that listeners, irrespective of their musical training and performance experience, perceived differences in the synchronization in the duo tokens depending on the presence/absence of visual contact between singers; on the other hand, the smaller asynchrony patterns measured across rehearsals in the singing quintet recordings were not perceived. This study contributes to our understanding of perceptions of synchronization by individuals with different levels of musical expertise, and underscores the perceptual salience of synchronization, regardless of musical experience.

## Introduction

Musicians strive to maintain a high level of synchronization with their co-performer(s) during ensemble performances, although timing varies within and between players, establishing small asynchronies between the performers [1]. Empirical investigations have been conducted to analyse the perceptibility threshold of asynchronies and the factors that might affect asynchrony perception. Wallach and his colleagues found that two identical sounds, of which one was delayed, were perceived as a single fused sound when presented in close succession [2]. This phenomenon, defined as the *precedence effect*, was found to vary by the type of sound; fusion occurred when the delay between the two sounds was up to 5 ms for short clicks, and up to 40 ms in the case of complex sounds, such as speech and music [2]. Research also shows that pure tones lasting 5 ms and temporally offset by 30 ms or more tend to be perceived as separate auditory streams [3]. Mean stream segregation ratings, which quantify the extent to which two tones are perceived within the same stream, are correlated with the tones’ frequency distance: the further apart their frequencies, the less likely they are to be heard in the same stream [3]. Listeners are more sensitive to detecting onset rather than offset asynchronies of multicomponent sounds, and perceive onset asynchrony more easily for complex sounds with components spaced harmonically rather than at equal intervals in logarithmic frequency, given the same number of components [4].

A few studies have investigated listeners’ discrimination of the temporal order of sonic events, analysing their ability to identify the correct order of two onsets, i.e. which onset came first. The threshold of asynchrony to detect the temporal order of two asynchronous tones for highly trained listeners is between 15 and 20 ms, and this is mostly independent of the frequency (high or low) and band (narrow or wide) of the sounds [5]. The temporal order discrimination threshold was further investigated by Pastore et al. [6], who demonstrated that it is strictly related to the stimulus duration, and can be as small as 5 ms if the duration of sinusoidal stimuli is as small as 10 ms. Recently, the hypothesis has been tested that the threshold for detection of asynchrony between complex music sounds such as piano tones will be higher than for steady-state sounds in case of highly trained musicians [7,8]. Researchers found that this discrimination depends on the tone type, improving with more artificial tones compared with acoustic piano tones. The threshold was around 20 ms for pure tones and around 30 ms for real piano tones spanning an interval of an octave or a major seventh, regardless of whether the pair began with the higher or lower tone. Research also found that this temporal order discrimination threshold was related to the music expertise of the listeners, reporting that nine musically untrained listeners (with zero to seven years of playing an instrument) were not able to discriminate the temporal order [9]. The research above identified the threshold of the temporal order discrimination in relation to pure tones and acoustic piano tones; discrimination in a more realistic scenario such as music ensembles is still to be investigated.

Research has also investigated listeners’ sensitivity to asynchrony in relation to the relative intensity of the sounds. The perception of major sixth dyads was tested by manipulating simultaneously the relative timing and intensity of two tones by ±54 ms and ±20 MIDI units [9,10]. Researchers found that the detection of asynchrony was less reliable when the louder upper tone entered before rather than after a softer, lower tone. They argued that this might be due to the effect of a forward masking, where a louder-anticipating sound masks a softer-following sound. In a later experiment, researchers investigated the influence of asynchrony and intensity on the perceptual salience of individual voices in multi-voiced musical context, comprising three-piano tone chords, sequences of piano chords, and the first nine bars of Chopin’s *Ballade* op. 38 [8,9]. Results show that intensity was the dominant cue, whilst the effect of note asynchrony on the perceived dominance of a particular tone was comparatively marginal.

In addition to the study of the perception of individual tones, research has recently investigated the perception of synchrony in the context of string quartet and jazz trio performances. Perception of between-player asynchrony variability, for example, has been analysed using computer-simulated string quartet performances of a short excerpt from Haydn op. 74 n. 1 [11]. Within-player timing variability and correction gain (i.e., the size of the adjustments relative to the asynchrony) were manipulated in two separate experiments. Results showed that listeners without a specialized music training were sensitive to the variability of note onset asynchrony and degree of correction gain, when judging the level of togetherness in quartet performances. This study identified the need for future research to consider variation in dynamics and more complex rhythms, since this might affect the perception of asynchrony variability. In addition to the study of the perception of synchronization in string quartets, a recent study has analysed the perception of synchronization of a pool of jazz trio recordings, comprising the original performance featuring asynchrony up to 26 ms, alongside recordings manipulated with increased and decreased asynchronies [12]. Results show that listeners, irrespective of their music training, preferred recordings with reduced asynchronies smaller than 19 ms over the original performance and the recordings manipulated to the largest asynchronies. This finding suggests that listeners might prefer performances containing asynchronies smaller than the perceptual threshold, but with natural timing variabilities given by the ensemble playing, which might make the performance distinguishable from that generated by a computer.

Research has also investigated the roles of music training on perceptual sensitivity to synchrony, providing complex results. As anticipated above, [9] found that musically untrained listeners were not able to discriminate the temporal order of pure tones, complex harmonic, or acoustic piano tones, while Wing et al. [11] reported that non-experts were sensitive to the degree of asynchrony when judging the level of togetherness in string quartet performances. Hofmann et al. [12] observed no effect of the expertise of the listeners (i.e., musicians, non-musicians, dancers) when rating their preferred jazz-trio performance from a pool of stimuli with manipulated asynchronies. More recently, it has been shown that the ability to detect asynchrony and discriminate temporal order at onsets can increase with multi-hour training in asynchrony and order tasks, and even a single exposure to such perceptual tasks can yield learning [13].

In summary, although asynchronies are inevitable during music performance and, to some extent, desirable, they are not always perceived by listeners. A number of factors can contribute to the detection of asynchronies, such as differences in frequency, duration, and sound intensity between two pure tones. The perceptual threshold is likely to increase with the complex onset behaviours of real music events, such as acoustic piano tones, and might change with the experience of the listeners. Recently, a few studies have investigated the role of visual contact and rehearsal on interpersonal synchronization between singers during singing ensemble performances. Research demonstrated that synchronization was better with visual contact between singers than without during singing duo performances [14], and improved between the first two rehearsals in a singing quintet rehearsing a homophonic piece across five rehearsal sessions during a four-month period [15]. However, whether listeners with different levels of expertise can perceive the effects of visual contact between singers and varying levels of rehearsal on the interpersonal synchronization between singers during ensemble performance has not yet been investigated.

This study aims to analyse whether listeners’ perceptions of synchronization reflect the different patterns of synchronization that were performed during ensemble performances with and without visual contact and across rehearsals, and whether listeners’ perceptions change with different levels of musical expertise. Specifically, this study addresses the following question:

- Do the differences in the asynchrony, physically measured in the singing ensemble performances in D’Amario et al. [14,15] and resulting from altered visual contact and degree of rehearsals, reflect the perception of synchronization by listeners with a variety of levels of musical experience?

Based on existing literature analysing the precedence effect among complex sounds [2], it was hypothesized that listeners might not perceive differences in the synchronization if the asynchronies were smaller than 40 ms, which is considered to be the threshold for the perception of complex sounds, such as speech and music. In other words, differences between asynchronies might not be perceived if the asynchronies in themselves are shorter than the threshold for perception.

## Methods

### Participants

Ethical approval for the study (with reference D’Amario211117) was obtained from the Physical Sciences Ethics Committee (PSEC) at the University of York (UK). Thirty-three participants (M = 25.2y, SD = 4.2; 15 females, 15 males, three non-binary) were recruited from the University of York (UK), comprising 10 undergraduate students, 16 postgraduate students, and seven doctoral researchers. Ten participants were from the Music Department, seven from the Psychology Department, and 16 from the Department of Electronic Engineering on Music Technology related programmes. Six students from the Department of Electronic Engineering were PhD students in the Audio Lab and had extensive experience in conducting perception studies not related to synchronization. As shown in Table 1, participants were classified based on their music training as non-experts and experts. The non-experts group comprised 20 university students with little or no music training. The experts group consisted of 13 advanced music students with at least four years of formal music training, and an average of 11.2 years formal training (SD = 4.7). The experts group comprised two sub-groups based on their performing experience: i) “performers in the study” group included five performers who performed the quintet recordings used as one subset of stimuli for the current study; and ii) the “other musicians” group comprised eight advanced music students. Based on their singing experience, the experts group was split into two more sub-groups: singers (seven) and instrumental players (six). The hearing of participants was not tested, but participants reported having normal hearing, except for three who reported sporadic tinnitus during their life that did not affect their hearing at the time of the experiment. Two participants reported having absolute pitch. They received a nominal fee of £5.

**Table 1:** Participant grouping based on music training, performing and singing experience.

Classification	Group (participants n)
Music training	Experts (13); Non-experts (20)
Experience performing in the study	Experts (13): Performers in the study (5); Other musicians (8)
Advanced singing *versus* instrumental experience	Experts (13): Singers (7); Instrumental players (6)

### Stimuli

**Fig 1:**
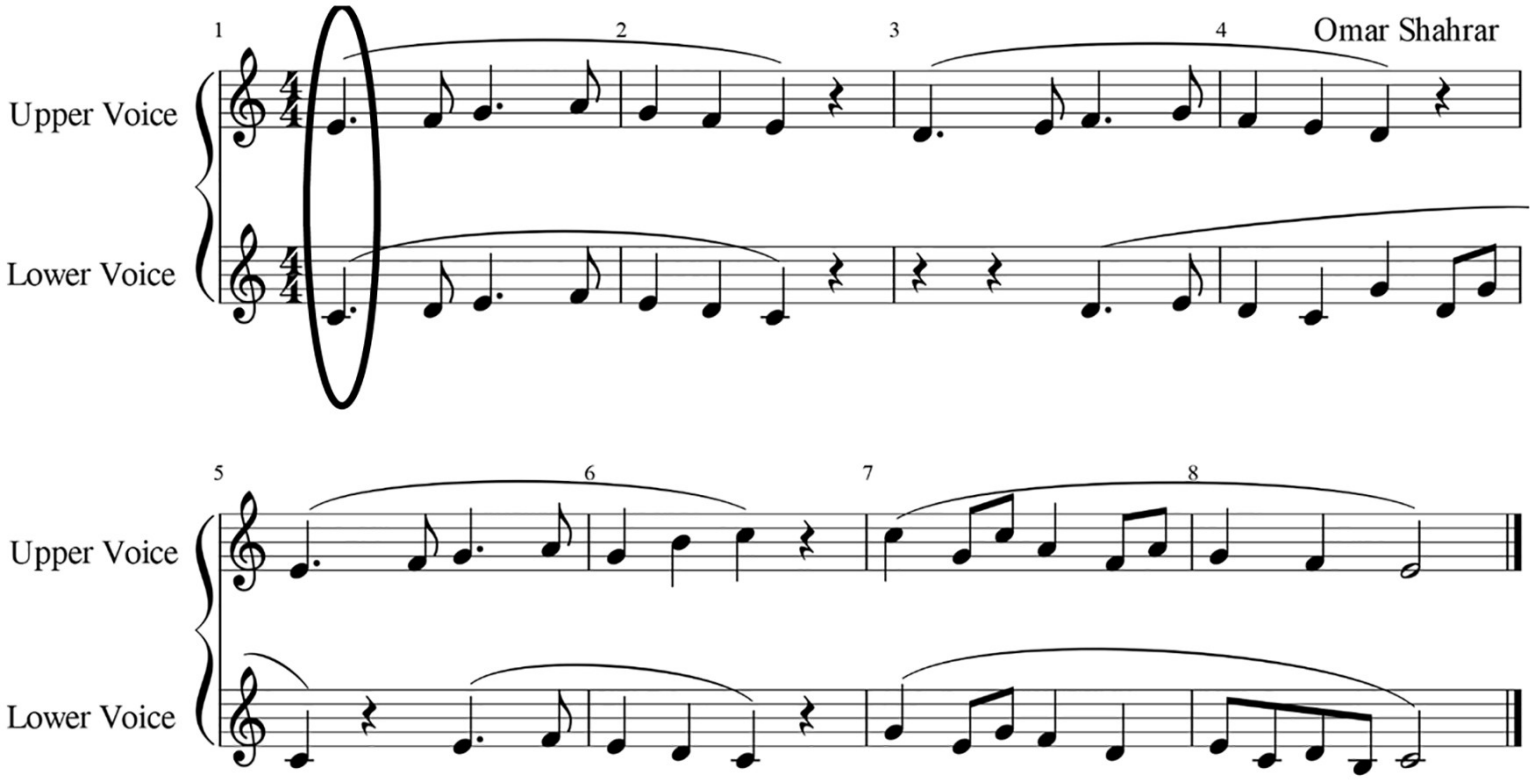
The score of a two-part piece composed for previous studies [14,16], highlighting the first note (circled) that was used for the perception study. **The authors retain copyright of this figure, under a Creative Commons Attribution License** (CC-BY).

Two sets of audio recordings were presented to the listeners, including 48 singing duo tokens and 10 singing quintet performances. The duo tokens (set A) were approximately 1 sec long and selected from a pool of 576 singing duo performances collected for a previous investigation, analysing the effect of visual contact on synchronization between singers during singing duo performances [14]. The duo recordings were performed by 12 semi-professional, newly-formed vocal duos singing the same, mostly homophonic, two-part piece composed for the study and shown in Fig 1. The piece was sung legato to the vowel /i/ to minimise onset variation, and electrolaryngographs (small electrodes placed either side of the larynx on the neck) were used to obtain and measure onsets and offsets of each note, as described in D’Amario et al. [14].

In D’Amario et al. [14], each duo repeated the same piece 24 times with visual contact between them, and 24 times without. In the present study, only the performance of the onset of the first note of the piece was used. This specific sound event was of great interest for perceptual analysis, since an effect of visual contact was found with the onset of the first note, and thus it was selected for the current investigation.

Precision of synchronization was computed calculating the absolute asynchronies at the onsets of the first note of each of the full data set of duo performances (i.e., 576 performances). Asynchrony was computed by subtracting the timing of one singer from the co-performer {for a full description, see D’Amario et al. [14]}; this step generated a list of 576 absolute asynchronies computed for the onset of the first note, half of which were performed in the presence of visual contact between singers and half without. A subset of 48 tokens of 1 sec was selected for the current perception study, to avoid fatiguing the listeners. To select the tokens, firstly, onsets were separately ranked for performances from smallest to largest asynchrony value with and without visual contact, resulting in two separate lists (i.e., one with and one without visual contact), each comprising 288 asynchronies. These two lists were then systematically sampled, choosing randomly the first asynchrony from each list and thereafter every 10th asynchrony. The fixed interval of 10 was arbitrarily decided to produce a total of 24 tokens for each condition (i.e., with and without visual contact). This sampling procedure produced two lists of 24 snippets with an unbalanced number of tokens for each duo arising from picking every 10th asynchrony among the 288 ranked tokens performed by all 12 duos. Each list was therefore amended whereby the absolute values of a given (over-represented) duo selected through the sampling process would be replaced with the equivalent absolute values of a duo not represented in the list, to create a final list that was balanced for duo as well as condition. This process assured that four tokens for each duo, two with and two without visual contact, were selected.

**Fig 2:**
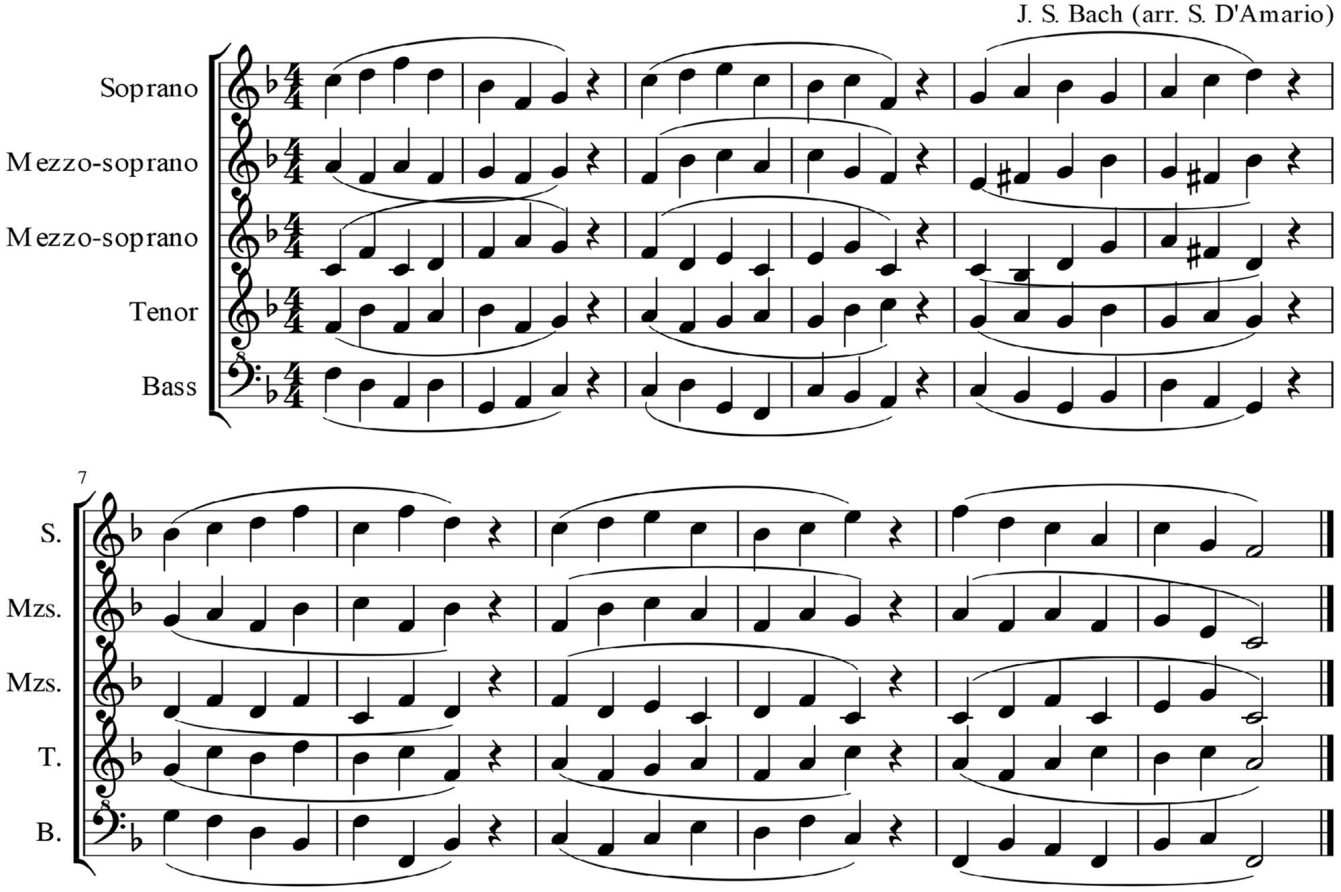
A singing quintet piece from a previous study [15], which was used in the current study. **The authors retain copyright of this figure, under a Creative Commons Attribution License** (CC-BY).

The other set of recordings, set B, included 10 quintet performances that were selected from a pool of 30 recordings of a clearly homophonic piece (see Fig 2), collected for a previous investigation analysing the evolution of synchronization during five rehearsals across a four-month study period [15]. The full data set comprised recordings of an advanced singing quintet who rehearsed the piece for 10 minutes in each rehearsal session, and performed three repetitions of the same piece before and after each rehearsal. This generated a total of six performances per rehearsal; each performance was approximately 50 sec long. As detailed in D’Amario et al. [15], precision of synchronization was computed calculating the absolute asynchronies for each pair of singers at onsets and offsets of phonation, and note beginnings and endings within a legato phrase for each performance. From the original data set, a list of 10 recordings was selected featuring the two most representative performances of each rehearsal based on the following three-step process:

1. The average asynchronies were computed for each performance/onset/offset/beginning/ending, across pairs of singers. This step produced a mean synchronization value for each onset/offset/beginning/ending of each performance/session, resulting in 84 mean asynchrony values for each performance comprising two values for each note (i.e. onset/note ending at beginning of phonation, note beginning/ending within a legato phrase, and note beginning/offset at the ending of phonation).
2. Using the list generated from step one, the average asynchrony for each performance was computed, pooling together the average values computed for each onset/offset/ending/beginning during the previous step. This process resulted in six mean asynchrony values (i.e., one per performance) for each rehearsal.
3. The grand means for each rehearsal were then calculated across the six mean values, and the two performances with the closest mean asynchrony values to the grand mean were selected for each rehearsal.

The stimuli were imported as .WAV files into the Qualtrics online platform (www.qualtrics.com), and were presented using a desktop PC computer and headphones (Beyerdynamic DT990) at a level of 80 dB, which was measured and setup through a polystyrene dummy head with no pinnae, housing a microphone (NTi M4260) connected to a sound level meter (NTi XL2). The audio of the recordings was manually equalized across recordings, but not within the same recording. Therefore, all recordings were heard at approximately the same volume, and the expressive dynamics of the performance remained unchanged.

### Design

As shown in Table 2, the study comprised two parts, namely Part A and Part B. Part A asked about the extent of synchronization in relation to the singing duo tokens (set A). Listeners were asked to answer the question: To what extent are the singers synchronized/together in time at the start of the following singing duo performances? Part B asked about the extent of synchronization in relation to the singing quintet performances (set B). Listeners were asked to answer the question: To what extent are the singers synchronized/together in time throughout the following quintet performances?

**Table 2:** Research questions, stimuli, and questions posed to listeners in the study.

Research questions	Stimuli	Listeners questions
RQ1: Do the differences in asynchronies resulting from altered visual contact reflect the perception of synchronization?	Set A: 48 singing duo tokens, ca 1 sec long	Q1. To what extent are the singers synchronized/together in time at the start of the following singing duo performances?
RQ2: Do the differences in asynchronies measured across five rehearsals reflect the perception of synchronization?	Set B: 10 singing quintet recordings, ca. 50 sec long	Q2. To what extent are the singers synchronized/together in time throughout the following quintet performances?

Parts A and B were presented in a counterbalanced order within each participant group (i.e., non-experts, performers in the study, and other musicians). The order in which the recordings were presented was randomized within each part for each participant.

### Procedure

Participants were invited to an individual laboratory session that took place in a quiet room at the Department of Electronic Engineering of the University of York (UK) in February 2018. Participants first gave written, informed consent to take part to the study, and answered a questionnaire regarding their demographic background, music training, and singing experience. Then, the two parts of the study and the related questions and stimuli were presented. Listeners were asked to rate the extent to which the singers were synchronized on a sliding scale from zero to 100. Zero indicated that the singers were not at all synchronized/together in time; 100 meant they were fully synchronized/together in time. Listeners were able to play the recordings within the same set as many times as they wished, and they could change their rating through the sliding scale if needed within the same set of recordings. Once one of the two parts of the set was completed, listeners were not able to rectify their ratings or play the recordings of that set again.

Participants were asked to take a five-minute break between the two parts to reduce fatigue. An artificial break was introduced after the first half of the quintet recordings with a reminder to judge the extent to which singers were synchronized/together in time throughout the quintet performances. The full experiment lasted approximately 45 minutes.

### Analysis

Stepwise multilevel linear-models of the response variable (i.e., perceived synchronization) were implemented to test the primary fixed effects of visual contact and rehearsal, and the random effects of participant. Listener groups based on expertise, performing or instrumental experience were also entered in the models as fixed effects nested within the primary fixed effects, as shown in Table 3. Multilevel linear models were chosen because they increase the statistical reliability of the fixed effects analyses assessing the inter-participant variation [17]. The models were implemented in R Studio [18] using the lme4 package.

**Table 3:** Primary and nested fixed effects on the perceived synchronization, and the related recording sets used in the multilevel linear models.

Model n	Primary fixed effects	Nested fixed effects	Recordings set
1	Visual contact	Expertise: experts (13), non-experts (20)	Set A: duo tokens
2	Visual contact	Instruments: singers (7), instrumental players (6)	Set A: duo tokens
3	Rehearsal number	Expertise: experts (13), non-experts (20)	Set B: quintet performances
4	Rehearsal number	Performing experience: performers in the study (5), other musicians (8), non-experts (20)	Set B: quintet performances
5	Performance asynchrony	Expertise: experts (13), non-experts (20)	Set A: duo tokens
6	Performance asynchrony	Instruments: singers (7), instrumental players (6)	Set A: duo tokens
7	Performance asynchrony	Expertise: experts (13), non-experts (20)	Set B: quintet performances
8	Performance asynchrony	Performing experience: performers in the study (5), other musicians (8), non-experts (20)	Set B: quintet performances

Perceived synchronization was the continuous response variable. Visual contact and rehearsal number were categorical explanatory variables with two (with and without visual contact) and five (rehearsal 1–5) categories, respectively. The nested fixed effect variables were also categorical variables. Performance asynchrony was a continuous explanatory variable.

The β coefficients on the predictor being considered are given below with reference to the specified base level of the factor, i.e. experts *versus* the base level non-experts, instrumental *versus* singing experience, and the “performers in the study” and “other musicians” groups *versus* the non-experts. The β-fixed effect coefficients indicate that for each one unit increment in the predictor being considered, the effect of the given predictor changes by the amount specified by the β coefficient.

Prior to the analysis of the perception of synchronization, the role of visual contact and rehearsal number on the precision of performed synchronization was analysed, based on the newly created subset of duo tokens and quintet recordings respectively, which were selected from the original data sets reported by D’Amario et al. [14,15] as explained in Methods/Stimuli. This was done to identify the main characteristics of the specific recordings used for the current perception study. As explained above, the sampling process used for the selection was done in such a way that the two subsets of data would be representative of the original, full data sets. Results from the subsets selected for the current study were expected to be similar but not exactly the same as those from the full data set. For this reason, it was important to analyse performed asynchronies of the data set of recordings selected for the current study. An independent *t*-test was conducted on the selected duo tokens to investigate whether there was a significant difference between the means of synchronies measured in the presence and absence of visual contact. A regression model was implemented to test the primary fixed effect of rehearsal number on the precision of synchronization computed across the quintet performances selected for the current study. Note number and category (i.e., note beginnings and endings within a legato phrase, and onsets and offsets of phonation) were entered into the model as random variables.

Perceived ratings that fell outside three times the interquartile range (IQR) were automatically identified as extreme outliers through SPSS (IBM SPSS Statistics v. 24) and excluded from the analysis.

A Bonferroni correction for multiple multilinear tests was implemented, and the alpha level was set at *p* = 0.005, based on a total of nine models developed in the study.

## Results

### Performance asynchrony

**Fig 3:**
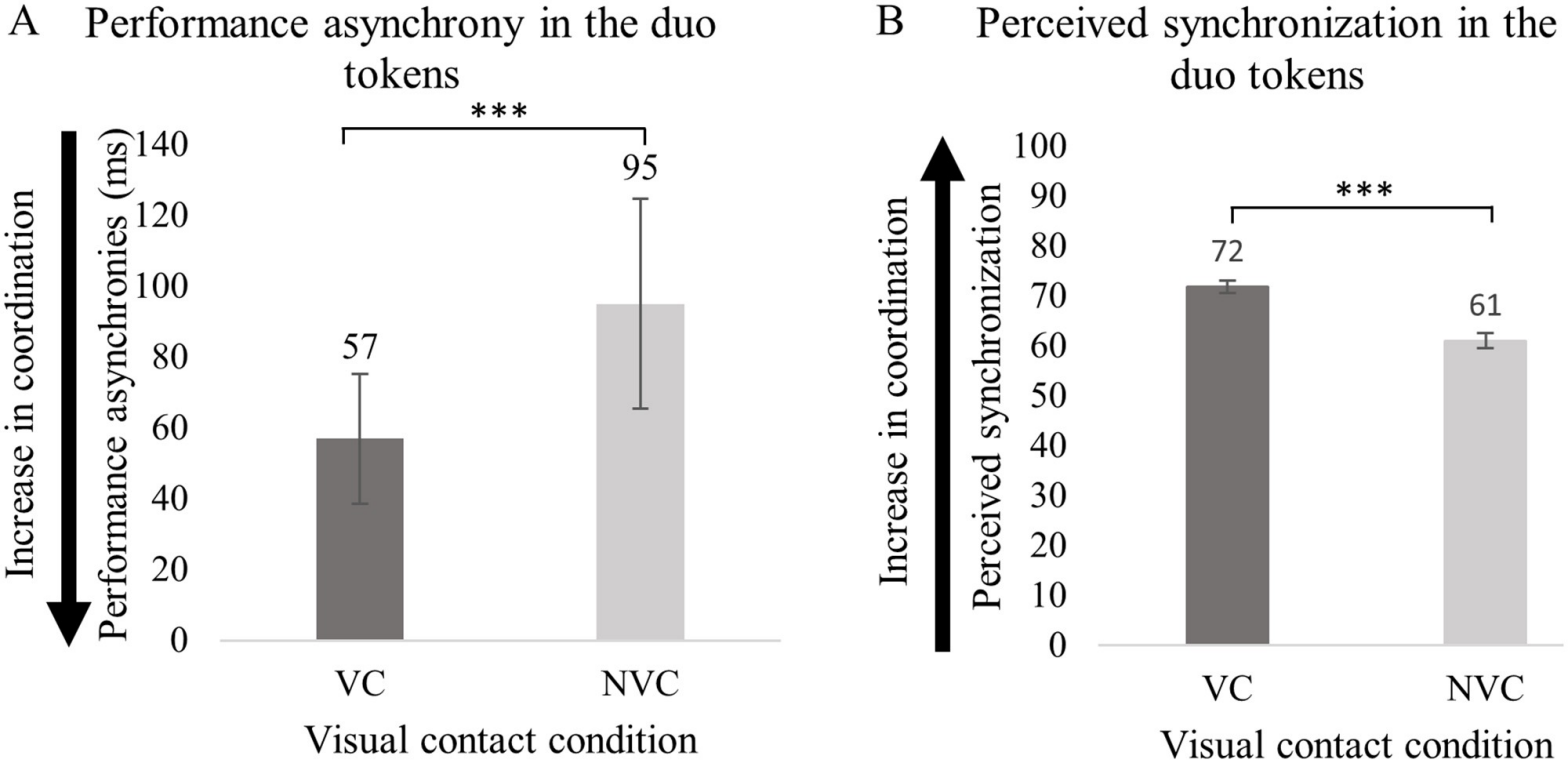
Synchronization in the duo tokens selected for the current study and performed in presence and absence of visual contact between singers. (A) displays the precision of the performed synchronization, and (B) ratings of perceived synchronization in a scale from zero to100, where zero indicates singers were “not at all synchronized/together in time” and 100 “perfectly synchronized/together in time”. Error bars represent 95% CI of the mean. *** *p* < 0.001.

Prior to the analysis of the perception of synchronization, an initial test was conducted to investigate the effects of the presence/absence of visual contact on the precision of synchronization, based on the subset of duo token data selected for this study from the previous investigation [14]. An independent *t*-test demonstrated that synchronization in the subset of data was significantly better in the presence of visual contact between singers (*M* = 57, *SD* = 44.9) than without it ⌊(*M* = 95, *SD* = 72.5), *t*(46) = −2.2, *p* < 0.001⌋, as shown in Fig 3A. The asynchronies measured with and without visual contact were on average 38 ms different; they were on average 57 ms with visual contact, and 95 ms without. As expected, this result corroborates the findings from the previous investigation based on the full data set of duo tokens showing that precision of synchronization was better with visual contact [14].

**Fig 4:**
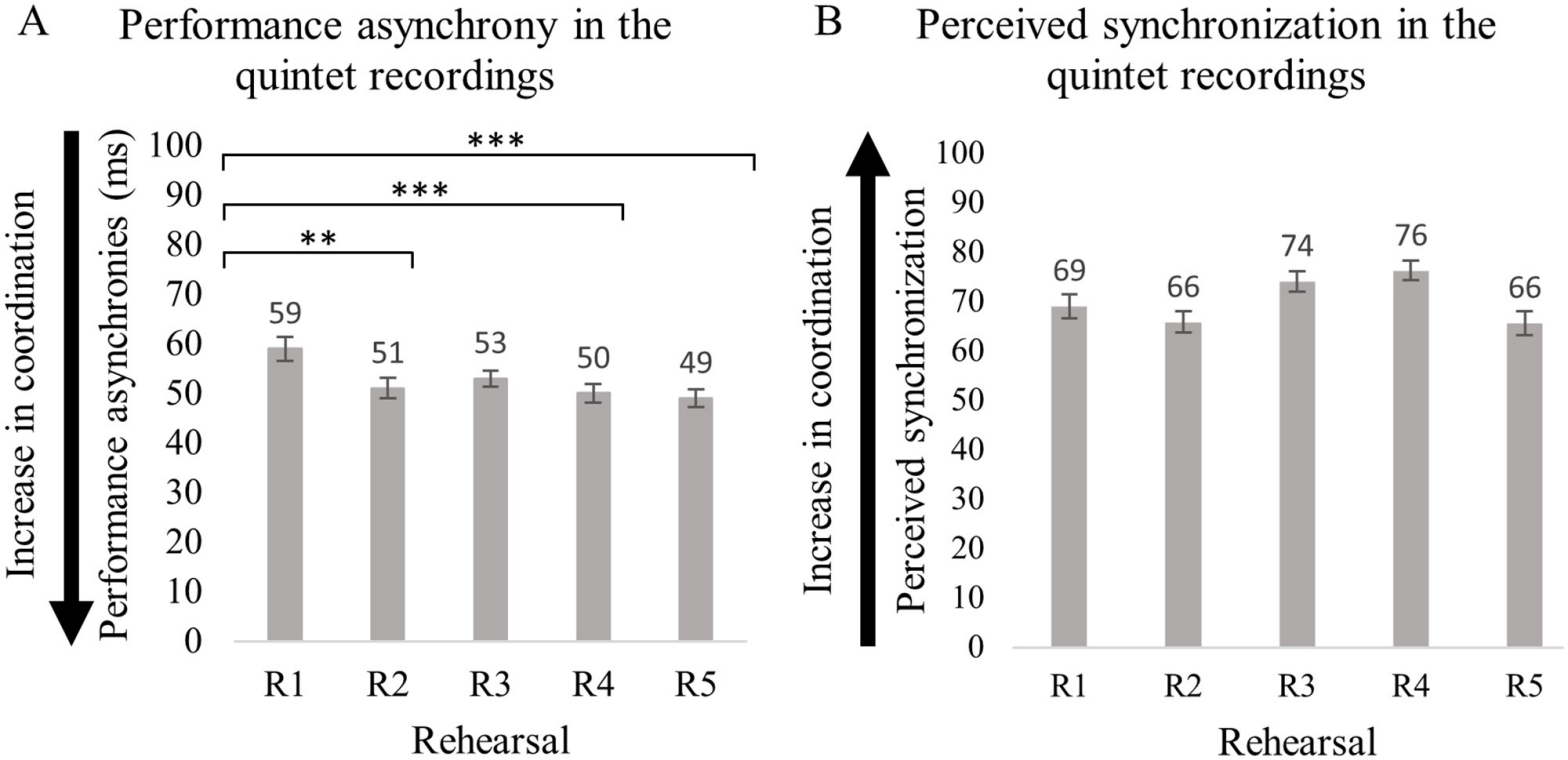
Synchronization in the quintet recordings selected for the current study and performed across five rehearsal sessions. (**A) displays the precision of the performed synchronization, and (B) ratings of perceived synchronization in a scale from zero to 100, where zero indicates singers were “not at all synchronized/together in time” and 100 “perfectly synchronized/together in time”**. Error bars represent 95% CI of the mean. *p*-values have been adjusted using the Holm method. ** *p* < 0.01, *** *p* < 0.001.

Results from the regression analysis conducted on the quintet recordings selected for the current investigation demonstrate that, compared with rehearsal 1, precision of synchronization improved in rehearsal 2 [*β*(−8.8), *t*(791.9) = −3.2, *p* < 0.01], in rehearsal 4 [*β*(−9.1), *t*(791.9) = −3.4, *p* < 0.01], and rehearsal 5 [*β*(−9.8), *t*(791.9) = −3.6, *p* < 0.001]. Post-hoc tests show no significant changes across rehearsals 3–5, as shown in Fig 4A. The variance partition coefficient (VPC) among note number and category was 0.1084 and 0.1325, respectively; this indicates that 10.8% and 13.3% of the variability of the effect of rehearsal on performed synchrony can be attributed to note number and category, respectively. The asynchronies measured on this subset of data were on average larger than 49 ms, and on average up to 10 ms different across the five-rehearsal sessions. As expected, these results are very similar to those based on the full data set collected for D’Amario et al. [15].

### Perceived synchronization

#### Visual contact

Results from the multilevel linear modelling (models n 1 and n 2, as shown in Table 3) of perceived synchronization related to singing duo recordings show that visual contact between singers predicted ratings of perceived synchronization [*β*(−8.8), *t*(1548) = −5.6, *p* < 0.001]. Listeners rated the recordings performed in the presence of visual contact between singers (*M* = 71.8, *SD* = 24.3) as being better synchronized than those performed without visual contact (*M* = 61.0, *SD* = 29.2), as shown in Fig 3B.

**Fig 5:**
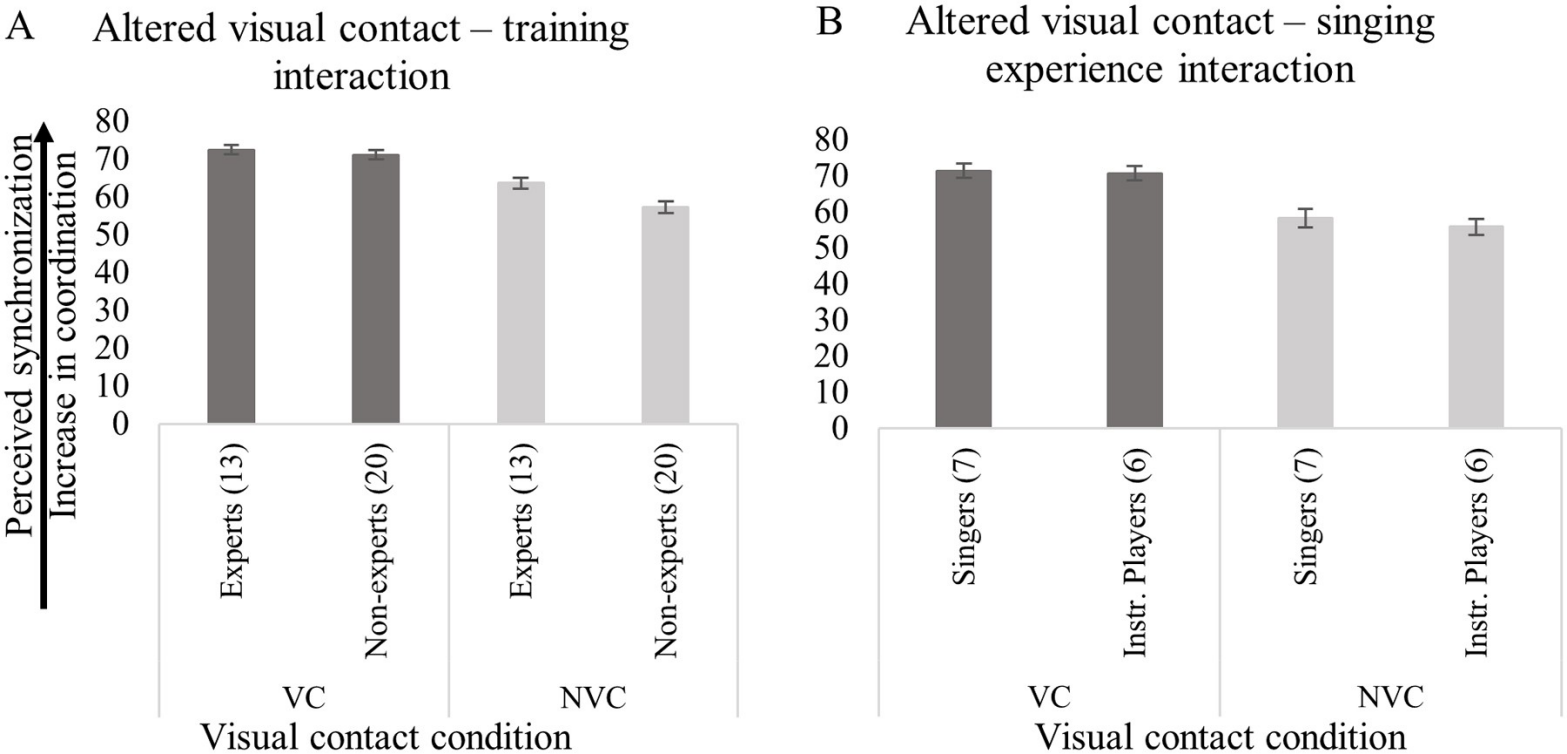
Perception of synchronization by: (A) interaction between presence/absence of visual contact and training level; and, (B) interaction between visual contact condition and singing experience. The number of listeners in each subgroup (i.e., experts, non-experts, singers, and instrumental players) is given (n). Error bars represent 95% CI of the mean.

Music training (i.e., experts *versus* non-experts) and instrumental experience (i.e., singers *versus* instrumental players) of respondents did not predict ratings of perceived synchronization based on the presence/absence of visual contact between singers, as shown in Fig 5A and 5B. The variance partition coefficient (VPC) among participants was 0.186, which indicates that 18.6% of the variability of the effect of visual contact on perceived synchrony can be attributed to the responded category.

#### Rehearsal number

The analysis of the effect of rehearsal (models n 3 and n 4, as shown in Table 3) demonstrates that rehearsal number (1–5) did not predict ratings of perceived synchronization (see Fig 4B). This is interesting in light of the synchronization measured in the recordings, highlighting an improvement in rehearsal 2, 4, and 5, compared with rehearsal 1, as show in Fig 5A.

**Fig 6:**
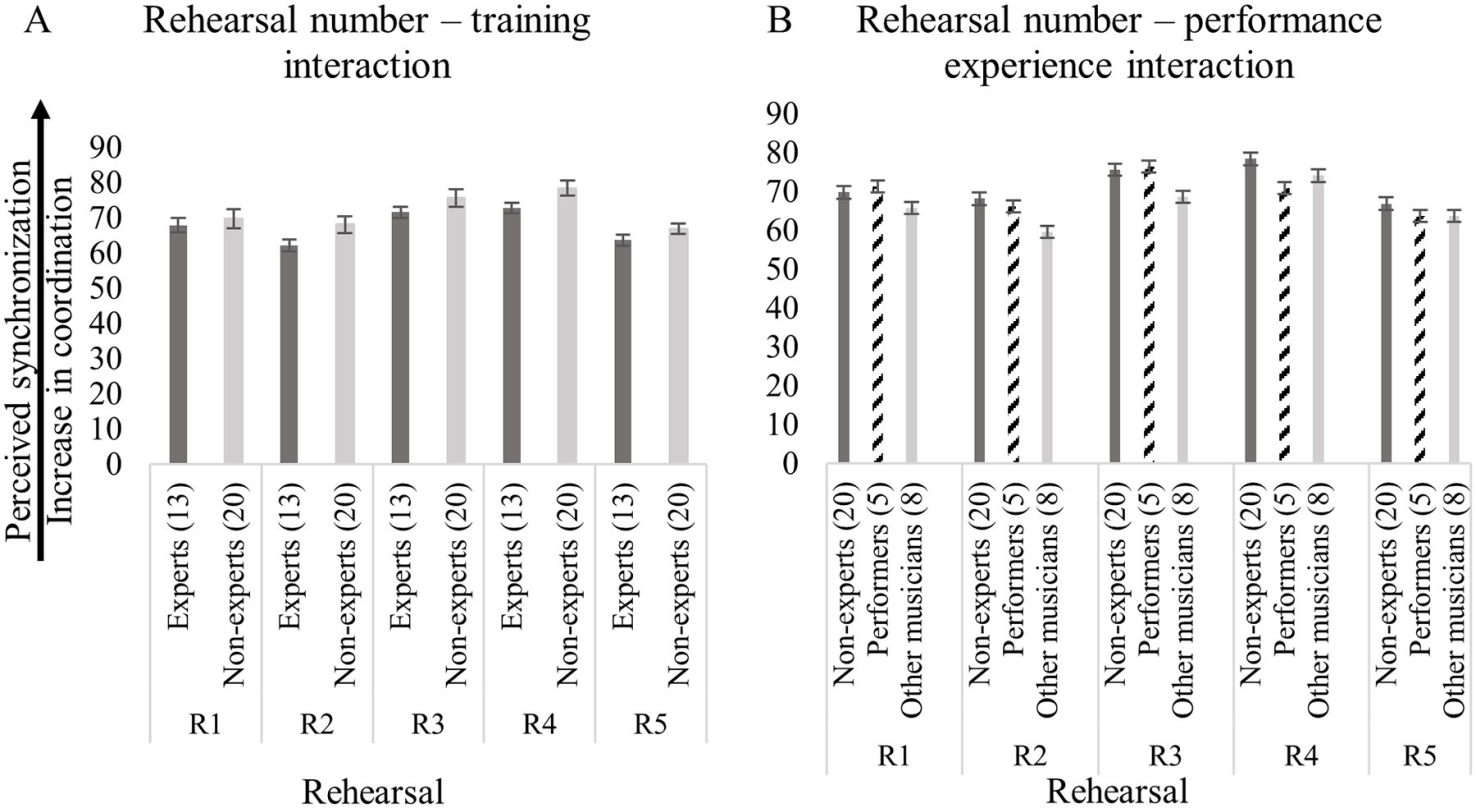
Perceived synchronization based on degree of rehearsal (from rehearsal 1, R1, to rehearsal 5, R5) in relation to: (A) the musical training of the listeners (i.e., non-experts, and experts); and (B) the performance experience of the listeners (i.e., non-experts, performers in the study, and other musicians). The (*n*) represents the number of listeners in each subgroup (i.e., experts, non-experts, performers in the study, and other musicians). Error bars represent 95% CI of the mean.

This result did not differ according to the listeners’ music training (i.e., experts *versus* non-experts) as shown in Fig 6A, or the performance experience of the listeners (i.e., non-experts, performers in the study, other musicians), as shown in Fig 6B. The VPC among participants was 0.42.

Precision of the quintet’s synchronization, indexed by absolute asynchronies, improved significantly in rehearsal 2, 4 and 5 compared with rehearsal 1, as shown in Fig 4B. Post-hoc tests were conducted to investigate whether listeners with different levels of training were able to perceive any differences between rehearsal 1–2, 1–4, and 1–5. This was not the case, irrespective of music training (i.e., experts *versus* non-experts) or performance experience of the listeners (i.e., non-experts, performers in the study, other musicians), as shown in Fig 6A and 6B.

### Relationship between performance asynchrony and perceived synchronization

Asynchrony measurements of the duo tokens and quintet recordings were entered stepwise in multilevel linear models (models n 5–8, see Table 3) to test whether the performance asynchronies predict the synchronization that listeners with different levels of expertise, training and performance experience can perceive.

**Fig 7:**
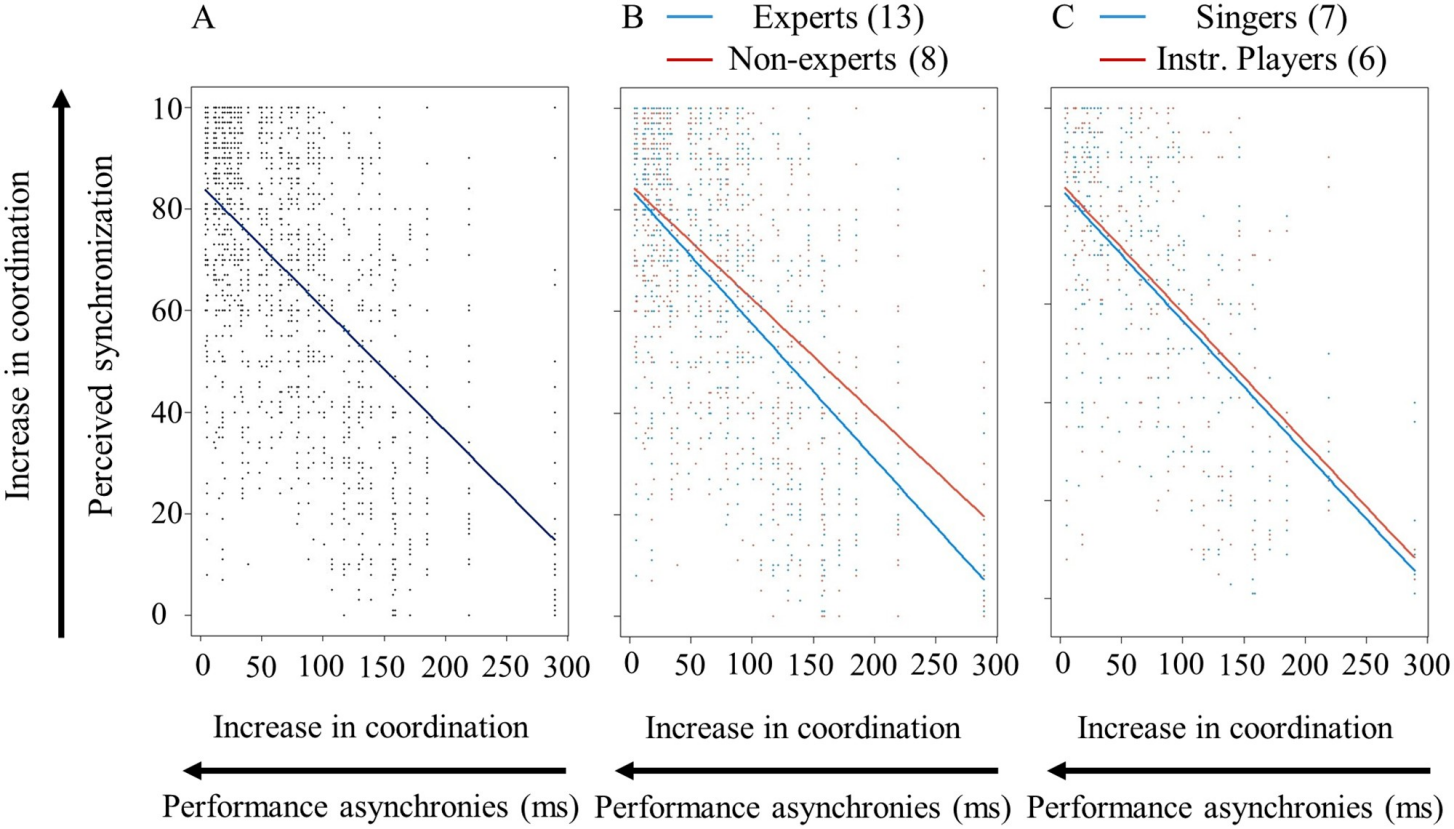
Relationship between performance asynchrony and perceived synchronization in the duo tokens. (A) displays the relationship between the performance asynchrony and the perceived synchronization; (B) shows the correlation between performance asynchrony and perceived synchronization in relation to the training of the listeners (i.e., experts and non-experts); and, (C) represents the correlation between performance asynchrony and perceived synchronization for singing and instrumental listeners.

Results show that there is a significant negative relationship between the performed asynchronies and the perceived synchronization of the duo tokens, as shown in Fig 7A. Specifically, one unit of reduced performance asynchrony (x-axis) is associated with an increase in perceived synchronization ratings (y-axis) by 0.23 units [*β*(−0.23), *t*(1573) = −22.2, *p* < 0.001]. In other words, listeners perceived the smaller performance asynchronies as being better synchronized than the larger performance asynchronies. Fig 7B illustrates the relationship between performance asynchrony and perceived synchronization based on the music training of the listeners. The blue line representing the experts’ ratings is steeper than the red one (non-experts’ rating), suggesting that experts might tend to rate larger asynchronies as being less synchronized than non-experts. This finding illustrates a general trend which fails to reach significance. In addition, the singing *versus* instrumental experience is not a significant predictor of the perceived synchronization, as shown in Fig 7C.

Results from the analysis of the quintet recordings demonstrate that the performance asynchronies measured across the five rehearsals did not predict ratings of perceived synchronization, irrespective of music training, and types of performance experience of the listeners.

## Discussion and conclusions

This study investigates the perceptions of performance synchronization by listeners with various forms of music expertise, in recordings with performance synchronization differences resulting from altered visual contact and five rehearsal sessions. The relevance of the different degrees of synchronization, physically measured in D’Amario et al. [14,15] through electrolaryngography, was considered from a perceptual perspective in the current study, as this is a concern for performers and audience alike. Participants listened to two sets of recordings, which were taken from D’Amario et al. [14,15], and which were representative of the findings from those studies. The sets of recordings comprised short duo tokens recorded with and without visual contact between singers, and full singing quintet performances recorded across five rehearsal sessions.

The duo tokens recorded in the presence of visual contact between singers were better synchronized than those without, as demonstrated by the analysis of the performance synchrony. The precision of synchronization, as quantified by the absolute asynchronies at onsets of phonation, was on average 57 ms in presence of visual contact between singers and 95 ms without. These differences in the performance synchrony were reflected in the perception of synchronization, irrespective of the music training and singing *versus* instrumental experience of the listeners. Listeners rated the tokens recorded with visual contact as better synchronized than those without visual contact. Specifically, one unit of reduced performance asynchrony was associated with an increase in perceived synchronization by 0.23 units.

The precision of synchronization in the singing quintet recordings selected for the current study was on average larger than 49 ms across rehearsals, and improved across the five rehearsals. Synchronization was better during the second, fourth and fifth rehearsal compared with the first. However, listeners were not able to perceive the significant differences in performance asynchrony that were measured across rehearsals, irrespective of the music training and performance experience of the listeners.

Listeners accurately perceived differences between performances sung with and without visual contact that differed on average by 38 ms, but they did not perceive differences between quintet performances that differed across rehearsals by only 10 ms on average. This suggests that listeners can perceive differences in asynchronies only above a certain threshold placed somewhere between 10 and 38 ms. These results complement the literature investigating the precedence effect, showing a threshold of asynchrony perception of around 40 ms for the perception of complex sounds, such as speech and music [2]. These findings also complement the more recent investigation conducted by Goebl and Parncutt [7] into temporal order discrimination, reporting a threshold of 30 ms for acoustic piano tones. Altogether, the results suggest that the threshold for asynchrony perception is around 40 ms for complex sounds; the threshold for the temporal order discrimination is around 30 ms for acoustic piano tones; and, the threshold to detect differences in asynchronies in singing ensemble recordings is between 10 and 38 ms.

The lack of differences in the perception of asynchronies that were on average up to 10 ms different might also be related to the complexities of recordings comprising five singers performing simultaneously. Differences in the perceptual demands of these two tasks (i.e., rating synchronization in duo tokens and full quintet recordings) might have affected the results. For a given temporal difference, the perception of asynchronies might be related to the number of tones, becoming more difficult as the number of simultaneous tones increases. Further investigations are needed to shed some light on the impact of the number of tones on the perception of synchronization.

Moreover, the length of the recordings might have also affected differences in the perception of such asynchronies. Pastore et al. [6] found that the temporal order discrimination was strictly related to the duration of the stimulus; it was as small as 5 ms if the duration of sinusoidal stimuli was as small as 10 ms, but the threshold was about 12 ms when the duration of the stimuli was 300 m. Similarly, the perceptual judgement of differences in the performance asynchrony in the current study might change based on the duration of the stimuli, and it might increase when the duration of the stimuli increases. Further investigations in this area are needed to investigate the perceptual threshold of the differences in relation to the length of the stimuli.

In addition, results from the current study expand findings of the perceptions of string quartet performances, showing that listeners without specific musical training were sensitive to the degree of asynchrony between performers when judging the level of togetherness in string quartet playing [11]. This study also further expands research on temporal order discrimination, which showed that non-experts were not able to discriminate the temporal order of pure and complex tones [9]. Altogether, these results suggest the musical training of the listeners might come into play for temporal order discrimination, but might not be relevant to the evaluation of the degree of synchronization in ensemble performances.

In the current study, the synchronization skills of the listeners were not systematically assessed; however, superior synchronization skills of the listeners might have affected the perceptions of synchronization. Further investigations should also consider the addition of a tapping task, to analyse the perceptual judgement of interpersonal synchronization in relation to the synchronization skills of the listeners.

A previous investigation showed that a single exposure to perceptual tasks judging synchronization and discriminating the temporal order of two tones can improve synchronization ability [13]. Further investigation might expand the current study investigating systematically the development of the listener’s synchronization skills through, for example, a training stage, to investigate whether perception of interpersonal synchronization in the context of ensemble performance improves with specific training. This aspect would be particularly important for musicians aiming to boost music performance.

Some duo tokens had audible breaths at the beginning of the snippet, which might have affected the ratings of synchronization, particularly as breathing has been identified as a ‘basic’ performance cue in ensemble performance with singers [19]. It would therefore be interesting in future research to investigate the extent to which audible breaths facilitate the perception of synchronization, particularly of singing.

Focussing on the singing voice in this study enabled a larger number of repetitions, as well as ensuring consistency in onset/offset detection through electrolaryngography. The nature of the individual onsets/offsets in terms of their acoustic characteristics, which could impact the perception of synchronisation, was not analysed here; this will likely vary to some degree across singers and repetitions. Future work considering the nature of onsets/offsets for synchronisation in singing would be a complex undertaking considering the difficulties in recording individual voices in a group setting, but could shed more light on the intricacies of synchronisation perception.

Perception of synchronisation may also differ across different instruments and with the addition of lyrics due to their characteristic onsets/offsets; similarly, perception at onsets may differ from that at offsets, since listeners are more sensitive to detecting onset rather than offset asynchronies of multicomponent complex sounds [4]. This study focused on a harmonic major-third dyad, taken from the beginning of the first note of a duet, and a clearly homophonic five-part piece sung legato to the vowel /i/, comprising onsets and offsets of phonation as well as note beginnings and endings within legato phrases. For greater musical validity, future investigations should explore the roles of various instruments, lyrics and onsets/offsets on perception of synchronization, and should involve the addition of timing complexities through rhythm and polyphony. The analysis of such features would also help to contextualise the findings of this study within a broader understanding of the role of synchronisation in musical listening and performance. Robust experimental investigation to specifically understand perceived asynchronies is needed before conclusions can be drawn, concerning overall judgments of performances by listeners within an ecological musical context.

In conclusion, this study showed that differences in the precision of synchronization measured in singing duo tokens in relation to altered visual contact were reflected in the perceptions of synchronization, irrespective of the music training and performance experience of the listeners. However, the smaller differences in performance asynchronies observed across rehearsals in the quintet recording were not reflected in the perceptions of synchronization, irrespective of the music training and performance experience of the listeners. This study provides a new context, from the perspective of perception, with which to consider the results of previous investigations [14,15], investigating the roles of altered visual contact and degree of rehearsal in interpersonal synchronization measured in the singing performances. Ultimately, the study has implications for musical performance as the findings suggest that the perceptual salience of synchronization is dependent on musical context, and might transcend the musical expertise of the listeners.
